# The Performance of the Vaginal Discharge Syndromic Management in Treating Vaginal and Cervical Infection: A Systematic Review and Meta-Analysis

**DOI:** 10.1371/journal.pone.0163365

**Published:** 2016-10-05

**Authors:** Charifa Zemouri, Teodora Elvira Wi, James Kiarie, Armando Seuc, Vittal Mogasale, Ahmed Latif, Nathalie Broutet

**Affiliations:** 1 Department of Reproductive Health and Research, World Health Organization, Geneva, Switzerland; 2 Department of Preventive Dentistry, Academic Center for Dentistry Amsterdam and University of Amsterdam and VU University, Amsterdam, The Netherlands; 3 Politic and Economic Research Center, International Vaccine Institute, Seoul, South Korea; 4 Public Health Consultant, Brisbane, Australia; University of Utah Health Sciences Center, UNITED STATES

## Abstract

**Background:**

This review aimed to synthesize and analyze the diagnostic accuracy and the likelihood of providing correct treatment of the syndromic approach Vaginal Discharge Flowchart in managing cervical infections caused by *Neisseria gonorrhoeae (NG)* and *Chlamydia trachomatis* (*CT)*, and vaginal infections caused by *Trichomonas vaginalis* (*TV)* and Bacterial vaginosis (BV) and *Candida albicans*. This review will inform updating the WHO 2003 guidelines on Vaginal Discharge syndromic case management.

**Methods:**

A systematic review was conducted on published studies from 01-01-2000 to 30-03-2015 in multiple databases. Studies evaluating the diagnostic accuracy and validation of the WHO Vaginal Discharge Flowchart were included. Validation parameters including sensitivity, specificity, positive predictive value (PPV) and negative predictive value (NPV) and the 95% confidence intervals for the different types of the flowchart were taken as outcomes, re-calculated, and analysed using a fixed model meta-analysis for data pooling. The level of agreement between the index and reference test were determined by the Cohen’s Kappa co-efficiency test. Each individual study was assessed on quality using the QUADAS-2 tool.

**Findings:**

The search yielded 2,845 studies of which 16 met the eligibility criteria for final analysis. The diagnostic performance to identify cervical infections was low and resulted in a high proportion of over and missed treatment. The four flowcharts had a sensitivity between 27.37% in history and risk assessment and 90.13% with microscopy, with the inverse in specificity rates. The treatment performances between the flowcharts were inconsistent. The same applies to the use of vaginal discharge flowchart for treating vaginal infections. For vaginal infections the vaginal discharge flowchart had a good performance in flowchart 3 with 91.68% of sensitivity; 99.97% specificity; 99.93% PPV and 0.02% who missed their treatment and 8.32% of women who were over treated by the vaginal discharge flowchart

**Conclusion:**

The vaginal discharge flowchart should focus on management of vaginal infection. It could be used as an intermediate approach for cervical infections for sex workers until a point of care test is available in resource poor settings.

## Background

Sexually Transmitted Infection (STI) case management is one of the top priorities in controlling STIs to break the chain of infection and transmission [1]. From 1984 to 1991 the World Health Organization (WHO) published the simplified (syndromic) approach based on field experience from countries such as Kenya, Swaziland and Zimbabwe [2]. Since then, based on available evidence, the WHO has updated the guidelines on syndromic case management [3, 4].

Syndromic case management provides a standardized evidence-based approach using clinical management algorithms, and flowcharts that can be used consistently across providers, so that primary healthcare providers in resource-poor settings may deliver appropriate and effective STI treatment. The treatment is based on the identification of consistent groups of symptoms and easily recognized signs (syndromes), risk assessment, and risk scoring [5, 6]. Syndromic management is widely utilized. In most resource poor settings these flowcharts are still the standard of care. Out of 109 countries with national STI treatment guidelines, 83 have adopted the STI syndromic case management approach [7]. In high and middle income countries results of laboratory tests for STI take some days to get back to the clinic, so even in these situations immediate treatment of symptomatic patients follows syndromic management guidelines.

Vaginal discharge is a common genital tract symptom among women. Studies have shown that of women seeking care in the primary and secondary health care setting, 11% to 38.4% in India, and 34% in Ethiopia were availing care for vaginal discharge[8–12]. Vaginal discharge may be a normal physiologic occurrence or a pathological manifestation. It is often challenging to distinguish abnormal from normal discharge, both from the patient’s and the health care provider’s perspective. Moreover, normal physiologic variations occur due to biological or hormonal changes [13, 14]. In India the symptom of vaginal discharge was also associated with psychosocial factors of non-infectious etiology [15]. A pathological vaginal discharge may be of vaginal or cervical origin. Discharge of vaginal origin may be associated with Bacterial vaginosis (BV), and infection with *Candida spp*. and *Trichomonas vaginalis* (TV). Discharge of cervical origin is usually due to infection with *Neisseria gonorrhoeae* (NG), *Chlamydia trachomatis* (CT), and *Mycoplasma genitalium* (MG); primary genital herpes simplex cervicitis can also manifest as vaginal discharge. Most cervical STIs do not cause any symptoms and syndromic management will not be able to identify or treat these infections unless the clinical management algorithm includes treatment for such infections. In order to detect specific pathogens causing cervicitis laboratory testing is necessary to identify the organisms involved.

The vaginal discharge flowchart leads to three treatment outcomes: no medical treatment; treatment for only vaginal infections caused by TV, BV and/or *Candida spp*. or treatment for vaginal, and cervical infections caused by NG and/or CT [3, 4]. In the majority of settings, abnormal vaginal discharge is highly indicative of a vaginal infection, thus all women presenting with vaginal discharge receive treatment for TV, BV and *Candida spp*. [4].

A series of evaluations of the syndromic management flowcharts was commissioned by the WHO and UNAIDS in the 1990s, and the results were published as a supplement in STIs in 1998. Of the 16 study sites, ten were in Africa. The main conclusions from these studies were that the flowcharts for urethral discharge and genital ulcer are reasonably sensitive and specific, whereas the flowchart for vaginal discharge is neither sensitive nor specific [16].

A review by Pettifor *et al* (2000) revealed that studies evaluating algorithms for vaginal discharge from 1993 to 1998 had sensitivities ranging from 73% to 93% among women presenting with symptoms of vaginal discharge and from 29% to 86% in women without symptoms [17]. The review also found that vaginal discharge is a poor indicator for cervical infection. It was recommended that risk assessment or risk scores can improve the efficacy for detecting NG and/or CT [3, 4]. Reviews of studies in 1990 suggest the potential for risk assessment to improve the sensitivity and reduce the cost per case treated compared with the pure symptom-based approach[17–20]. Another review published in 2000, by Sloan *et al* (2000), on the utility of syndromic approaches to screen for NG and/or CT in women showed that aggregated data of risk factors, risk scores, simple laboratory diagnosis and algorithms combining risk factors and speculum examinations are not effective approaches to identify or manage these conditions [21]. It should be noted that the majority of the earlier studies utilized cultures and Enzyme linked immunoassay (EIA) as gold-standard tests for NG and CT infections, which could have resulted in an underestimation of the diagnostic accuracy of the flowcharts. It was in 1998, when the more reliable PCR test was utilized as a gold standard test. All of these studies call for the need for point of care (POC) test to diagnose NG and CT. Progress has been made in the development of POC test for NG and CT, and it is a matter of time when these tests will be accessible widely and available in low and middle income countries. In the meantime the majority of countries continue to rely on syndromic case management.

This systematic review examines studies from 2001 onwards to ascertain information on the diagnostic accuracy and the likelihood of providing correct treatment (treatment performance) of the vaginal discharge flowchart in managing cervical infections caused by NG and CT and vaginal infections caused by TV and BV. Candidiasis will be excluded for analysis since it is not considered a STI and a part of the resident flora. This review will inform updating the WHO 2003 guidelines on Vaginal Discharge syndromic case management.

## Methods

### Electronic search and study selection

This study is set up based upon the PRISMA guidelines, see S1 Checklist. We searched the literature using the PRISMA guidelines for relevant articles using search terms such as “vaginal discharge” and “flowchart” throughout: PubMed; Cochrane Library; EMBASE; Global Health Library; and POPLINE from January 1, 2001, to March 30, 2015. The detailed search strategy is shown in Appendix 1. The review only included studies published from January, 2001 onwards as the WHO syndromic management guidelines were developed in 2001.

Studies that evaluated the diagnostic accuracy and validation of the WHO vaginal discharge flowchart compared to any laboratory diagnostic test and were methodologically sound were included. Studies that did not distinguish between cervical infection (NG and CT) and vaginal infections (TV and BV) were not included in the final review. Studies that presented data on sensitivity, specificity, positive predictive values (PPV), negative predictive value (NPV) or that provided data from which these parameters could be calculated using two by two tables were included. We excluded studies published in languages other than English, French, Spanish and Dutch. Case reports and letter to editor were excluded. The search hits were entered in EndNote Web. T.W. and C.Z. assessed the studies for relevance, title, abstract, and content and applied the inclusion criteria to the full text articles. In case of disagreement between the reviewers, a discussion followed in order to reach consensus, otherwise a third independent person was consulted.

### Data extraction and management

For each included study we used a standardized form to extract the data on: author, year of publication, study design, sample size, study population, characteristics of participants, STI prevalence rates, type of flowchart used, laboratory diagnostic tests used, sensitivity and specificity rates. Additional information on the limitations of the study, risk assessments of the flowcharts and risk of bias are ascertained to evaluate results and for discussion.

### Reference and index tests

All flowcharts (the index tests) had an entry point of women complaining of vaginal discharge followed by history taking including risk assessment to verify the presence of vaginal discharge. Flowcharts were categorized based on the following: Flowchart 1 = history and risk assessment; Flowchart 2 = history, risk assessment and speculum examination; Flowchart 3 = history, risk assessment, speculum examination, and vaginal discharge samples for Gram staining and microscopy; Flowchart 4 = country adapted flowcharts or those not defined by the study method. The majority of the country adapted flowcharts had risk factors that were specific for individual country context.

We defined persons with the actual conditions to be positive for the gold standard tests (reference test) defined as: nucleic acid amplification test (NAATs) for CT, NG, and TV; culture for NG and TV; and Nugent or Amsel Criteria (clue cells+, vaginal pH whiff test and presence of homogenous discharge) for BV [13,22, 23]. We did not include *Candida spp*. as one of the infection causing vaginal discharge, since the common symptom of candidiasis is vulvo-vaginal itchiness rather than vaginal discharge [21].

### Diagnostic accuracy

Data on the diagnostic accuracy which included: sensitivity, specificity, PPV and NPV was taken directly from the source paper or calculated from the data provided by using two by two tables. The diagnostic accuracy of the different flowcharts to identify persons with cervical infections: NG and CT and the vaginal infections: TV and BV are shown in Table 1. These results, in turn, were used to calculate the proportion of women that were provided with correct treatment, missed treatment and overtreatment. We estimated correct treatment rate as the proportion of patients correctly identified as requiring treatment or not; over treatment rate as proportion of non-infected patients who received treatment; and missed treatment rate as the proportion of infected patients who did not receive treatment.

**Table 1:** Measures used to determine the diagnostic accuracy.

Syndromic Approach: Vaginal Discharge Flowchart	Positive according to lab.	Negative according to lab.	Total	
**Positive according to flowchart.**	True Positives (TP)	False Positives (FP)	TP+FP	**PPV:** TP/(TP+FP)
**Negative according to flowchart.**	False Negatives (FN)	True Negatives (TN)	FN+TN	**NPV:** TN/(FN+TN)
**Total**	TP+FN	TN+FP	Sample Size (N)	
** **	**Sensitivity:** TP/(TP+FN)	**Specificity:** TN/(FP+TN)		
** **	**Missed treatment:** 1-sensitivity	**Over treatment:** 1-specificity	**Correct treatment:** (TP+TN)/Nx100	

### Statistical analysis

We conducted a meta-analysis by fixed effect model with pooling of samples from all studies within different types of flowcharts. We calculated the pooled sensitivity, specificity, PPV and NPV and the 95% confidence intervals for the different type of the flowcharts using the WINPEPI version 11.50 (August 2015). If the study had presented the results separately for NG, CT, TV and BV, the study with the higher PPV was included in the meta-analyses so as not to over represent any study.

The Cohen’s Kappa co-efficiency test, using the Landis & Koch (1977) cut-off points for kappa values, were applied to determine the level of agreement of the flowchart with the gold standard laboratory diagnostic test were used. We considered a flowchart to be useful when κ = > 0.21, and preferred values closer to 1 which indicates perfect agreement with laboratory diagnostics.

### Quality assessment

We assessed the risk of bias of the different studies using the QUADAS-2 assessment tool [22]. We graded as high, low and unclear the risk of bias in terms of patient selection, index test, reference standard and timing and the applicability concerns in terms of patient selection, index test and reference standard.

## Results

### Study selection

**Fig 1:**
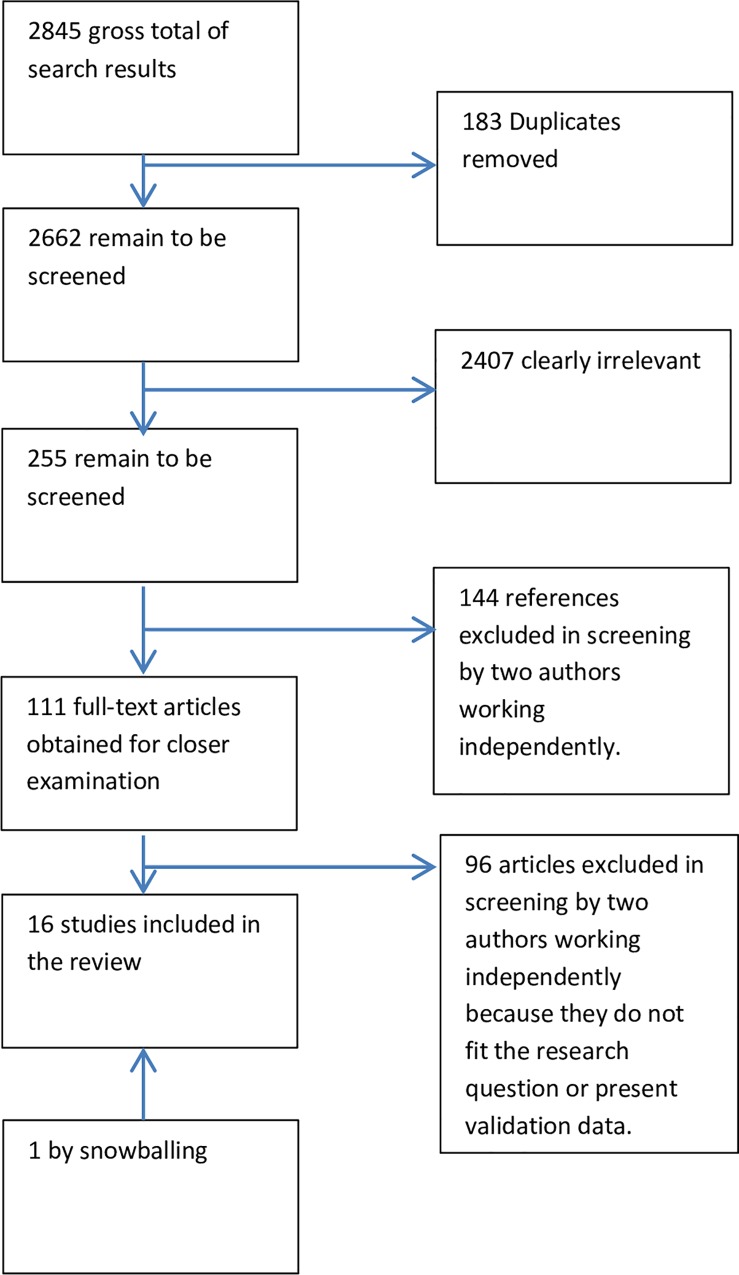
PRISMA flowchart depicting screening process.

The search strategy has resulted in 2,845 studies, of these 183 were duplicates and 2,407 were irrelevant. Irrelevant studies were those that did not fit the subject of our research, e.g. vaginal discharge in animals or flowcharts for medication prescriptions. A further 239 studies were not available in full text and could not be provided by the library nor by contacting the authors. This may be a potential for publication bias and should be taken into account. Sixteen studies were selected in the final review [23–39]. These studies distinguished between cervical infections due to NG and CT and vaginal infection due to TV and BV during their research and analysis. See Fig 1 PRISMA flowchart for the study selection.

### Study characteristics

Fifteen studies are cross-sectional studies and one study is case control. A total of 10,538 women participated in the studies. Their mean age was 27 years (range 14 to 67 years). For cervical infection 4,200 women were analyzed; 4,040 for vaginal infections and 3,556 for both infections. Studies took place in Africa (N = 6), South America (N = 6), Asia (N = 2), Europe (N = 2). Eight studies were conducted among general population women, five among pregnant women, one among adolescents and two among sexworkers. An overview of the study characteristics are summarized in Table 2.

**Table 2:** Study characteristics of studies included in the analysis.

Study	Country	Design	N	Prevalence (%)	Setting	Population	Flowchart	Reference test
Clark 2009	Peru	Cross-sectional	320	NG: 2.8 CT: 14.1	General health clinic	General population women	WHO 1	NG/CT: NAAT
Cornier 2010	Bulgaria	Cross-sectional	424	NG: 0.7 CT: 9.2 TV: 2.9 CT/NG: 9.5	Sexual health clinic	Non pregnant women	WHO 1,2,3, MSF 1	NG/CT: NAAT BV/TV: Microscopy
Das 2011	India	Cross-sectional	417	NG: 14.1 CT: 17.1 TV: 31.1 BV: 71 NG/CT:26.1	STI clinic for sex workers	Sex workers	WHO1,2 NACO 3	NG/CT: NAAT TV: PCR BV: Nugent’s criteria
Desai 2003	India	Cross-sectional	118	NG: 15.3 CT: 8.5 TV: 14.4	Red light district	Sex workers	NACO 2	NG: Culture and Gram staining CT: Pace 2 CT assay. TV: Wet mount
Francis 2014	Tanzania	Cross-sectional	966	NG: 4 CT: 12 TV: 19	Women working in bars, hotels.	HIV negative women	WHO 2	NG/CT: PCR TV: culture BV: Nugents criteria
Garcia 2004	Peru	Cross-sectional	754	NG: 1.2 CT:6.8	Mothers Club	General population	Peruvian Algorithm 1	NG/CT: PCR
Kisa 2009	Turkey	Cross-sectional	300	TV: 14	Maternal health clinic	Married women	WHO 2	TV: Wet mount
Lima 2013	Brazil	Cross-sectional	104	TV: 3.8 BV: 27.9	ANC	Pregnant women	WHO 1	TV: wet mount BV: Amsel criteria.
Msuya 2009	Tanzania	Cross-sectional	2645	TV: 5 BV: 20.9 Either. 23.9	ANC	Pregnant women	Tanzanian STI case management 2	TV: Wet mount BV: Amsel Nugent.
Moherdaui 2005	Honduras	Cross-sectional	933	NG/CT: 5.9 TV: 6.8 BV: 27.4	General health clinic	General population	WHO 1,2,3	NG: Gram CT: immuno-florence TV: microscopy
Onyekownu 2011	Nigeria	Cross-sectional	195	NG/CT: 12.8 BV/TV: 57.4	STI Clinic	General population	Nigeria National Algorithm (2b)	NG: Culture. CT: Elisa. TV: wet mount BV: Nugents criteria
Rassjo 2006	Uganda	Cross-sectional	199	NG: 9 CT: 4.5	Youth health clinic	Adolescents	National Algorithm 2	NG/CT/TV: PCR.
Romoren 2007	Botswana	Cross sectional	703	NG: 3 CT: 8 TV: 18.8 BV: 38.1	ANC	Pregnant women	WHO 2	NG/CT: LCR TV: wet-mount BV: Nugents criteria
Smith Fawzi 2006	Haiti	Case- Control	944	NG: 1.7 CT: 6.2 either: 7.4	Women's health clinic	General population	WHO 1, 2, 3, Haiti National Algorithm 1	NG/CT: Gen Probe PACE 2
Tann, 2006	Uganda	Cross-sectional	250	TV: 17.3 BV: 47.7	ANC	Pregnant women	Nigeria National Algorithm 2	TV: inoculation culture media kit & wet mount BV: Nugents criteria.
Tolosa, 2012	Colombia	Cross-sectional	1266	NG: 1.2 CT: 9 TV: 0.9 BV: 39	General health clinic	General population	WHO 1	NG/CT: PCR TV: wet mount BV: Nugents criteria

*Abbreviations*: ANC = Antenatal clinic; BV = Bacterial Vaginosis; CT: *Chlamydia trachomatis*; HIV = Human Immunodeficiency Virus; LCR = Ligase Chain Reaction; NAAT = Nucleic Acid Amplification Test; NG: *Neisseria gonorrhea*; PCR = Polymerase Chain Reaction; TV = *Trichomonas vaginalis*; WHO = World Health Organization; NACO: National AIDS Control Organization

### Vaginal Discharge Flowcharts

Three studies validated all types of vaginal discharge flowcharts for cervical infection—NG and CT, and two studies for vaginal infection—TV and BV. All studies compared the diagnostic performance of the Vaginal Discharge Flowchart with a laboratory reference test: six studies on cervical infections, and three on vaginal infections compared the flowchart with gold standard laboratory diagnostics, others tested vaginal samples with other types of laboratory diagnostics such as ELISA, microscopy or Gram staining.

### Cervical infections

Prevalence rates vary per study, NG ranged from 0.7% in Bulgaria [24] to 15.3% in India [26]. CT ranged from 5.9% in Honduras [32] to 17.1% in in India [25]. The diagnostic validity of the different types of vaginal discharge flowcharts to identify NG and CT is summarized in Table 3.

**Table 3:** Diagnostic performance of different vaginal flowcharts for treating NG and/or CT.

Flowchart 1													
Study	Gold Standard	Flow-chart	NG Prev (%)	CT Prev (%)	Sens (%)	Spec (%)	PPV (%)	NPV (%)		Over treatment (%)	Missed treatment (%)	Correct treatment (%)	K
Clark 2009	YES	1	2.8	14.1	(25/52) 48.1	(119/268) 44.4	(25/174) 14.4	(119/146) 81.5		55.6	51.9	45	0.039
Cornier 2010	YES	1	9.5		(25/40) 62.5	(230/383) 60	(25/178) 14	(230/245) 93.9		39	37.5	60.3	0.089
Das 2011	YES	1	26.1		(74/109) 67.9	(114/308) 37	(74/268) 27.6	(114/149) 76.5		23.5	72.4	45.1	0.249
Moherdaui 2005	NO	1	4		(6/60)16.2	(842/896) 94	(6/60) 10	(842/873) 96.5		6	83.8	90.9	0.079
Smith Fawzi 2002	YES	1	7.4		(24/69) 34.8	(553/870) 63.6	(24/341) 7	(553/598) 92.5		36.4	65.2	61.5	0.006
Tolosa 2012	YES	1	1.2	9	(14/127) 11	(1030/1133) 90.9	(14/117) 11	(1030/1143) 90.1		9	89	82.9	0.020
**Flowchart 2**													
Cornier 2010	YES	2	9.5		(37/40) 92.5	(107/383) 27.9	(37/313) 11.8	(107/110) 97.3	72		7.5	34	0.050
Das 2011	YES	2	26.1		(91/109) 83.5	(66/308) 21.4	(91/333) 27.3	(66/84) 78.6	21.43		72.67	37.65	0.22
Francis	YES	2	4	N/A	(12/92) 13	(2015/2185) 92.2	(12/182) 6.6	(2015/2095) 96.2	7.8		87	89	0.036
Francis	YES	2	N/A	12	(20/183) 10.9	(1934/2096) 92.3	(20/182) 11	(1934/2097) 92.2	7.7		89.1	85.8	0.032
Francis	YES	2	11.33		(32/258) 12.4	(1869/2019) 92.6	(32/182) 17.6	(1869/2095) 89.2	7.4		87.6	83.4	0.057
Moherdaui 2005	NO	2	4		(19/40) 48.7	(505/953) 53	(19/468) 4.1	(505/525) 96.2	47.0		51.3	56.2	0.001
Romoren 2007	YES	2	3	8	(2/11) 16.7	(79/93) 85	(2/16) 12.5	(79/88) 88.8	15.1		83.3	77.9	0.014
Rassjo 2006	YES	2	11.5		(14/23) 60.9	(68/176) 38.6	(14/122) 11.5	(68/77) 88.3	61.4		39.1	41.2	0.002
Smith Fawzi 2002	YES	2	7.4		(27/69) 38.6	(510/870) 58.6	(27/387) 7	(510/552) 92	41.4		61.4	57.2	0.009
Flowchart 3													
Cornier 2010	YES	3	9.5		(39/40) 97.5	(50/383) 13.1	(39/372) 10.5	(50/51) 98	87		2.5	21	0.022
Moherdaui 2005	NO	3	4		(21/37) 57	(537/895) 60	(21/379) 5.5	(537/553) 97.1	40		43.2	59.8	0.031
Smith Fawzi 2002	YES	3	7.4		(48/70) 68.6	(271/874) 31.0	(48/651) 7.4	(271/293) 92.5	69		31.4	33.8	0.001
Flowchart 4													
Cornier 2010	YES	MSF 1b	9.5		(34/40) 85	(150/383) 39.2	(34/267) 12.7	(150/156) 96.2	60.8		15	43.5	0.068
Das 2010	YES	Flow chart 2 + gram stain	26.1		(93/109) 85.3	(58/308) 18.8	(93/343) 27.1	(58/74) 78.4	21.6		72.9	36.2	0.025
Desai 2003	NO	NACO 2b	N/A	8.5	(7/10) 70	(54/108) 50	(7/61) 11.5	(54/57) 94.7	50		30	51.7	0.06
Desai 2003	NO	NACO 4 2b	10.2	N/A	(7/12) 58.3	(52/106) 49.1	(7/61) 11.5	(52/57) 91.2	50.9		41.7	50.00	0.026
Desai 2003	NO	NACO 4 2b	20.3		(13/24) 54.2	(46/94) 48.9	(13/61) 21.3	(46/57) 80.7	51.1		45.8	50	0.020
Garcia 2004	YES	Peru algo	1.2	N/A	(3/9) 33.3	(579/743) 77.9	(3/167) 1.8	(579/585) 99	22.1		66.7	77.4	0.012
Garcia 2004	YES	Peru algo	N/A	6.8	(17/51) 33.3	(573/701) 73.9	(17/145) 11.7	(573/607) 94.4	18.3		66	78.5	0.084
Garcia 2004	YES	Peru algo	7.45		(20/60) 33.3	(545/692) 78.8	(20/167) 12	(545/692) 78.8	21.2		66.7	75.1	0.064
Onyekownu 2011	NO	NNA	12.8		(5/25) 20	(156/170) 91.8	(5/19) 26.3	(156/176) 88.6	8.2		80	82.6	0.131
Rassjo 2006	YES	1b risk score	11.5		(6/23) 26.1	(119/176) 67.6	(6/63) 9.5	(119/136) 87.5	32.4		73.9	62.8	0.036
Smith Fawzi 2002	YES	HNA	7.4		(66/68) 97.1	(131/856) 15.3	(66/791) 8.3	(131/1393) 98.5	84.7		2.9	21.3	0.021

NG: *Neisseria gonorrhoeae;* CT: *Chlamydia trachomatis;* PPV: positive predictive value; NPV: negative predictive value; Prev: prevalence; Sens: sensitivity; Spec: specificity; K: Kappa value; N/A: Not applicable NNA: Nigerian National Algorithm; HNA: Haiti national algorithm; NACO: National AIDS Control Organization

The pooled sensitivities, in Table 4, were low for flowchart 1 (27.4%), and 2 (37.4%) while 3 and 4 had better sensitivity of 90.1% and 83.6% respectively. This is inversely proportional with specificity, which revealed sensitivity of 84.9% for flowchart 1, 79% for flowchart 2, 35.3% for flowchart 3 and 45.3% for flowchart 4. In the three studies that compared the diagnostic accuracy of the syndromic case management, the sensitivity increased with the addition of speculum examination and microscopy but specificity decreased. Studies with a combined prevalence of less than 20% had a PPV consistently below 10%. Higher prevalence resulted in higher PPV. Studies conducted among sex workers [25, 26] had PPV of above 20%. Flowchart 1, 2 and 3 have high NPV above 90%, however flowchart 4 had a NPV of only 42.5%. All flowcharts showed to have a poor agreement with laboratory diagnosis, with Kappa values varying from κ = 0.000 to κ = 0.061.

**Table 4:** Pooled diagnostic validity- Cervical infection.

Fixed effect model–inverse-variance estimates					
Flowchart	N studies	Sensitivity	Specificity	PPV	NPV
**1**	6	27.4 (23.9–30.9)	84.9 (83.9–85.9)	12 (10.2–13.9)	93.5 (92.6–94.3)
**2**	7	37.4 (36.9–2.9)	79 (78–79.9)	8.3 (7.1–9.5)	92.23 (91.4–93.1)
**3**	3	90.1 (85.8–94.4)	35.3 (33.4–37.1)	7.3 (6–8.7)	96.5 (95.3–97.7)
**4**	7	83.92 (80.9–7)	45.3 (43.9–47.9)	11.6 (10.1–13.1)	42.5 (41.3–43.6)
		**Missed treatment**	**Over treatment**	**Correct treatment**	
**1**	6	75.82 (72.5–79.1)	13.6 (12.9–14.6)	77.8 (76.6–78.9)	
**2**	7	70.7 (68–73.5)	18.5 (17.5–19.4)	69.2 (68–70.3)	
**3**	3	9.9 (5.6–14.2)	64.7 (62.9–66.6)	40.2 (38.2–42.1)	
**4**	7	36 (33.2–38.7)	50.5 (49–52)	47.6 (46.1–49.1)	

The low diagnostic performance of the different flowcharts to identify NG and CT resulted in a substantial proportion of overtreatment and missed treatment, and a relatively lower proportion of correct treatment. Studies with high sensitivity had low rates of missed treatment, but generally have low specificity with concomitant high rates of overtreatment. Studies with high specificity resulted in a higher proportion of cases with infection and without infection treated correctly. Flowchart 1 and 2 are more efficient since more cases were correctly treated (77.8% and 69.2% respectively) compared to 3, and 4 (40.2% and 47.6%). Flowcharts 1, and 2 have lower rates of overtreatment (13.6% and 18.5%) but resulted in more cases that had missed treatment (75.8% and 70.7%). Flowchart 3, and 4 had overtreatment of 64.7%, and 50.5% with fewer missed treatments (9.9% and 36% respectively). Flowcharts that have been adapted to the specific country context (flowchart 4) performed better compared to flowchart 2, and 3.

### Vaginal infection

Prevalence rates vary per study: TV ranged from 0.9% in Colombia [39] to 17.3% in Uganda [38]. BV ranged from 39% in Colombia [39] to 47.7% in Uganda [38]. The diagnostic accuracy rates and performance per flowcharts for identifying TV and BV are summarized in Table 5.

**Table 5:** Diagnostic performance of vaginal discharge for treating BV and/or TV.

Flowchart 1												
Study	Gold Standard	Flow-chart	NG Prev (%)	CT Prev (%)	Sens (%)	Spec (%)	PPV (%)	NPV (%)	Over treatment (%)	Missed treatment (%)	Correct treatment (%)	K
**Das 2014**	YES	1	71	31.1	(188/308) 61	(39.4/94) 41.9	(188/243) 77.4	(39/159) 24.5	75.5	22.6	56.5	0.025
**Garcia 2004**	NO	1	48.9		(96/369) 26	(308/385) 80	(96/173) 55.5	(308/581) 53	20	74	53.6	N/A
**Kisa 2009**	NO	1	27.8	16.7	(110/120) 91.5	(125/180) 69.7	(110/164) 67.1	(125/136) 91.9	30.6	8.3	78.3	N/A
**Lima 2013**	YES	1	27.9	N/A	(29/29) 100	(48/75) 64	(29/56) 51.8	(48/48) 100	36	42.2	74	0.239
**Lima 2013**	NO	1	N/A	3.8	(2/4) 50	(46/100) 46	(2/56) 3.6	(46/48) 95.8	54	50	46.2	N/A
**Lima 2013**	NO	1	31.7		(31/33) 93.9	(46/71) 64.8	(31/56) 55.4	(46/48) 95.8	35.2	6.1	74	N/A
**Romoren 2007**	YES	1	38.1	N/A	(50/268) 18.7	(365/435) 83.9	(50/120) 41.7	(365/583) 62.6	16.1	81.3	59	0.033
**Romoren 2007**	NO	1	N/A	18.8	(28/132) 21.2	(480/571) 84.1	(28/119) 23.5	(480/584) 82.2	15.9	78.8	72.3	N/A
**Romoren 2007**	NO	1	51		(69/359) 19.2	(296/344) 86	(69/117) 59	(296/586) 50.5	14	80.8	51.9	N/A
**Tann 2006**	NO	1	47.7		(58/116) 50	(68/127) 53.5	(58/117) 49.6	(68/126) 54	46.5	50	51.9	N/A
**Tann 2006**	YES	1	17.1		(28/42) 66.7	(113/203) 55.7	(28/118) 23.7	(113/127) 89	44.3	33.3	57.6	N/A
**Tolosa 2012**	NO	1	48.2		(497/608) 81.7	(221/652) 33.9	(497/928) 53.6	(221/332) 66.6	66.1	18.3	57	N/A
**Flowchart 2**																										
Cornier 2010	NO	1.2	35.7		(150/150) 100	(3/270) 1.1	(150/417) 36.1	(3/3) 100	98.9	0	36.4	N/A
Garcia 2004	YES	2	30.6	N/A	(110/229) 48	(189/519) 36.4	(110/440) 25	(189/308) 61.4	63.6	52	40	0.124
Garcia 2004	NO	2	N/A	16.5	(74/124) 59.7	(405/627) 64.6	(74/296) 25	(405/455) 89	35.4	40.3	63.5	N/A
Garcia 2004	NO	2	48.9		(179/369) 48.5	(268/385) 69.6	(179/296) 60.5	(268/458) 58.5	30.4	51.5	59.3	N/A
Francis 2014	YES	2	45.8	N/A	(150/1819) 8.2	(2010/2149) 93.5	(150/289) 51.9	(2010/3679) 54.6	6.5	91.8	54.4	0.032
Francis 2014	NO	2	N/A	19	(43/365) 11.8	(1840/1914) 96.1	(43/117) 36.8	(1840/2162) 85.1	3.9	88.2	82.6	N/A
Francis 2014	NO	2	48.4		(89/1142) 7.8	(1126/1219) 92.4	(89/182) 49.9	(1126/2179) 51.7	7.6	92.2	51.5	N/A
Moherdaui 2005	NO	2	27.4	6.8	(146/318) 46	(381/615) 62	(146/380) 38.4	(381/553) 68.9	38.1	54.1	56.5	N/A
**Flowchart 3**																										
**Cornier 2010**	NO	3	35.7		(141/150) 94	(197/270) 74	(141/214) 66	(197/206) 96	27	6.00	80.5	N/A
**Moherdaui 2005**	NO	3	27.4	6.8	(286/318) 90	(615/615) 100	(286/286) 100	(615/647) 95.1	-	10.1	96.6	N/A
**Flowchart 4**																										
Cornier 2010	NO	MSF 1b	35.7		(132/150) 88	(254/270) 94.1	(132/148) 89.2	(254/272) 9.4	5.9	12	91.9	N/A
Desai 2003	NO	NACO 2b	N/A	14.4	(15/17) 88.2	(55/101) 54.6	(15/61) 24.6	(55/57) 96.5	45.5	11.8	59.3	N/A
Msuya 2009	NO	TNA	N/A	5	(37/129) 28.7	(2076/2525) 81.5	(37/486) 7.61	(2076/2525) 95.8	17.8	71.3	79.6	N/A
Msuya 2009	NO	TNA	20.1	N/A	(136/533) 25.5	(1771/2121) 83.5	(136/486) 28	(1771/2168) 81.7	17.3	74.5	71.5	N/A
Msuya 2009	NO	TNA	23.9		(160/611) 26.2	(1717/2043) 84	(160/486) 32.9	(1717/2168) 79.2	16	73.8	70.7	N/A
Onyekownu 2011	NO(tv) YES (bv)	NNA	57.4		(98/112) 87.5	(5/83) 6	(98/176) 55.7	(5/19) 26.3	94	12.5	52.8	N/A

NG: *Neisseria gonorrhoeae;* CT: *Chlamydia trachomatis;* PPV: positive predictive value; NPV: negative predictive value; Prev: prevalence; Sens: sensitivity; Spec: specificity; K: Kappa value; N/A: Not applicable; TNA: Tanzanian Algorithm; NNA: Nigerian National Algorithm; MSF: Medicin Sans Frontier; NACO: National AIDS Controlling Organization.

The vaginal discharge flowchart had a better diagnostic accuracy for TV and BV compared to the flowchart used for cervical infections. The pooled sensitivity for flowcharts 2 and 3 had a sensitivity around 90%, while 1 and 4 were around 55%. Pooled specificity ranged from 41.39% to 99.97%. The PPV for identifying vaginal infection was higher compared to identifying NG and CT with flowchart 1 at 56.7%, flowchart 2 at 44.1%, flowchart 3 at 99.9% and flowchart 4 at 53.8%. The summary of the pooled diagnostic validity is summarized in Table 6.

**Table 6:** Pooled diagnostic validity–Vaginal infection.

Fixed effect model–inverse-variance estimates						
Flowchart	N		Sensitivity	Specificity	PPV	NPV
**1**	7	59.7 (57.9–61.4)		63.8 (612–65.4)	56.7 (54.5–58.8)	66.4 (64.6–68.2)
**2**	4	93.3 (92.9–93.7)		41.4 (40.5–42.3)	44.1 (41.4–46.8)	56.21 (54.5–57.9)
**3**	2	91.7 (89.2–94.2)		100 (99.9–100)	99.9 (99.7–100)	95.2 (94–96.6)
**4**	4	53.9 (51.3–56.5)		80.6 (79.2–81.9)	53.8 (51–56.6)	83.7 (82.2–85.1)
		**Missed treatment**		**Over treatment**	**Correct treatment**	
**1**	7	38.5 (36.8–40.3)		38.4 (36.5–40.1)	58 (56.5–59.5)	
**2**	4	1.1 (0.9–1.2)		58 (57.1–59)	52.4 (51–53.8)	
**3**	2	8.3 (5.8–10.8)		0.0 (0–0.1)	95.2 (94.0–96.3)	
**4**	4	46.1 (43.5–48.7)		19.5 (18.1–20.8)	75.8 (74.4–77.2)	

Flowchart 3, which consisted an additional simple laboratory test for TV and BV resulted in 95.2% correct treatment, a small proportion of missed treatment (8.3%) and almost no overtreatment (0.02% 95CI 0–0.1%). Flowcharts 1 and 2 resulted in correct treatment of 58% and 52.4%, respectively. Using flowchart 1 missed and over treatment occurred in 38% of the analyzed women, while the addition of a speculum examination (flowchart 2) did not improve the proportion of correct treatment and has led to a higher proportion of over treatment from 38.4% to 56%. Using specific national flowcharts resulted in 75.8% of women being correctly treated, 46.1% missed treatment and 20% received an over treatment for TV and BV.

Studies showing a good agreement according to the Kappa test, are consistent in reporting higher sensitivity, specificity, PPV, NPV, and correct treatment rates. Four flowcharts had a fair to perfect agreement with the laboratory diagnostics, described in Table 5: (i) India, Desai *et al* 2003, flowchart 4: κ = 0.21; (ii) Turkey, Kisa *et al* 2009 flowchart 1: κ = 0.264; (iii) Bulgaria, Cornier *et al* 2010, flowchart 3: κ = 0.597; and (iv) Honduras, Moherdaui *et al* 2005 flowchart 3: κ = 0.871.

### Risk of bias

Six out of 16 studies had risk of bias for patient selection; two for index test; seven for reference standard; and one for flow and timing. Based on the overall criteria for risk of bias, 72% had low, 27% with high, and 7% with unclear risk of bias. The applicability of test in terms of index test and reference standards is generally good. The details of the risk of bias and study applicability assessment are shown in Table 7.

**Table 7:** Risk of bias and applicability.

	PATIENT SELECTION	INDEX TEST	REFERENCE STANDARD	FLOW AND TIMING
Clark 2009	Unclear	Low	Low	Low
Cornier 2010	Low	Low	Low	High
Das 2011	Low	Low	Low	Low
Desai 2003	High	High	High	Low
Francis 2014	Low	High	Low	Low
Garcia 2004	High	Low	Low	Low
Kisa 2009	High	Low	High	Low
Lima 2013	High	Low	High	Low
Moherdaui 2005	Low	Low	High	Low
Msuya 2009	Low	Low	High	Low
Onyekownu 2011	Low	Low	High	Low
Rassjo 2006	High	Low	Low	Low
Romoren 2007	Low	Low	Low	Low
Smith Fawzi 2006	Low	Unclear	Low	Unclear
Tann 2006	Low	Low	High	Low
Tolosa 2009	High	Low	Low	Unclear

## Discussion

The review revealed that the diagnostic accuracy of vaginal discharge flowchartto identify NG and CT performs poorly across all studies. The Cohen’s Kappa values to identify cervical infection did not have agreement with the laboratory tests. The review also showed that the diagnostic accuracy of vaginal discharge flowchart to identify TV and BV was much better. Studies conducted among general population women with lower prevalence of NG and CT had consistently low PPV below 10%. Overall the flowchart utilized for high prevalence setting like among sex workers [25, 26]with more than 20% NG and CT rates had PPV above 20% and better diagnostic accuracy, but still performs poorly. The PPV of majority of the flowchart were above 50%, with more cases correctly treated. This is consistent with other reviews [17, 40, 41]. The diagnostic accuracy of the flowchart is associated with the prevalence rates of infection. The lower the prevalence rate, the lower the PPV, which in turn results in more false positive cases. A PPV of 10% translates to about 90% of cases identified by the flowchart to be positive do not have the infection. The prevalence of NG and CT among sex workers was more than 20% in our study and this was associated with a higher PPV over 20%. In high prevalence settings, the flowcharts performed better and could be cost effective. Flowcharts of high sensitivity will detect more NG and CT and will have an overall impact in reducing transmission. In this setting overtreatment will be acceptable. Utilizing the vaginal discharge flowchart to identify NG and CT among sex workers could also be an opportunity for promotion of condom use and screening for cervical infection [6]. The prevalence of NG and CT in the general population including pregnant women is low and hence the PPV of the flowchart will also be low [6, 42, 43], with consequent high rates of false positivity and overtreatment. This not only increases the overall cost of treatment and potential side effects, but also the psychological cost, break up of relations, and stigma. Several studies have recommended that women in low prevalence settings should not be treated for cervical infections [14, 35, 43, 44]. Previous reviews and studies showed poor correlation between vaginal discharge and NG and CT [6, 14, 23, 36, 40, 44, 45]. This is consistent with our findings that women who complain of vaginal discharge symptoms often do not have a cervical infection. In addition, most cervical STIs do not cause any symptoms and this will not be identified by syndromic approach. Additional symptoms are not recognized or not acted upon [46, 47]. The treatment results and the flowchart accuracy rates in our review provides us with the same conclusion, which makes our results consistent with others.

In the majority of cases of vaginal discharge the cause was either TV or BV. The prevalence of TV and BV among women with vaginal discharge was high and was associated with a concomitantly high PPV for identifying vaginal infection. Previous studies which analysed the association between the presence of vaginal discharge and vaginal infections caused by TV, BV showed a significant association with odds ratios from 3 to 7 [28, 43]. The Cohen’s Kappa values for four flowcharts have a fair to perfect agreement with the laboratory tests, which confirms the positive utility of the flowchart. The vaginal discharge flowchart seems to be an accurate tool based on our review. These results are also confirmed in previous studies in both the general population and sex workers [6, 14, 22, 24, 29]. However, the algorithm seems to be less effective in pregnant women complaining of vaginal discharge [14, 48]. We found the same conclusion in an analysis of our study by Tann *et al*, 2006, where the accuracy and correct treatment rates are around 50% [38]. It could be that vaginal discharge in pregnant women is mainly due to candidiasis [21]. The lack of agreement of the flowchart in pregnant women is confirmed by the Cohen’s Kappa score of κ = 0.130 in BV and κ = 0.154 in TV, which tells us that women are diagnosed by chance. However, there are issues of bias given that most of the comparison laboratory tests are not gold standard test.

The addition of speculum examination to identify cervical and vaginal infection increased the sensitivity of the flowcharts at the expense of specificity resulting in increased overtreatment and decreased correct treatment for both cervical and vaginal infections. Speculum examination is a standard care practice and this seems to be easy, but will require resources and may not be feasible in some settings.

The addition of microscopy to determine the presence of pus cells as a surrogate for cervical infection had increased the sensitivity of the flowchart at the expense of specificity, resulting in overtreatment and less cases that are correctly treated. These algorithms had the highest cost per true NG/CT case treated due to more resources required for speculum examination and laboratory diagnosis and higher drug cost due to overtreatment. In contrast, the addition of microscopy to identify TV and BV improved the diagnostic accuracy which could result in more cases being correctly treated, and marked reduction in overtreatment and missed treatment.

Applying a risk assessment can increase the sensitivity of the flowchart, so that more women with cervical STIs are correctly identified. The gain in sensitivity comes at the expense of specificity, which increases the number of women treated inappropriately. Risk assessment should be context and country specific, however studies needed to determine the appropriate risk assessment questions may not be able to be carried out in all countries, or areas within a country. Commonly, risk assessments questions developed in one setting are utilized in other settings. Most of these risk assessments include a question about new recent or multiple sexual partnerships. Questions that are appropriate for women attending STI clinics cannot be transferred directly to settings in which STI are rare and sex before or outside marriage can be severely punished, such as in the Middle East [49]. Acceptability is a major issue for implementing risk assessment in practice. Risk assessment questions that are reported to be acceptable during the development and piloting phases might not be workable in practice [20]. Questions that are perceived as intrusive or time-consuming are difficult to use as the entry point for a syndromic algorithm at two levels, healthcare workers who feel uncomfortable asking the questions will not ask them and women who are asked will not answer them [49].

### Effectiveness and cost effectiveness of Syndromic Approach

Several studies have demonstrated the effectiveness of syndromic approach. A RCT conducted in West African countries that implemented syndromic management for vaginal discharge have shown that treatment for TV and BV through a single dose regimen compared to a multi dose regimen was equally effective [48]. Implementation of syndromic approach in South Africa from 1995 to 2005 reduced the prevalence of syphilis, NG, BV, and substantially decreased chancroid. The effect of syndromic approach was more evident among symptomatic cases compared to asymptomatic cases [50]. A reduction of STIs syndromes was observed since its introduction in 1995 in Kenya but increased again in 2001 due to the termination of free medicines [51].

In addition to the diagnostic accuracy of syndromic approach, other factors such as the stability of risk factors, the health care providers implementing the approach, acceptability of stakeholders and availability of resources are important considerations in the effectiveness of syndromic approach. A study conducted in Botswana public health clinic, evaluating the quality of syndromic management of STI revealed that a third of women did not receive appropriate treatment based on the syndrome and that there is a need to improve the quality of medical history taking and clinical examination [52]. White et al. estimated that overall, only 13% of symptomatic curable STI episodes have been cured through syndromic treatment in a rural town in Africa. Since the introduction of syndromic approach in 1995, curable STIs remain to be prevalent and it has been suggested that this could be improved by increasing rates of treatment seeking and provision of correct treatment [53].

A cluster randomized trial has shown that the comprehensive syndromic management package, which includes condom promotion and supply, partner notification, and STI advice, increased provision of STI information in women, and the cost per patient appropriately managed was USD 1.51, the study concluded that STI syndromic package improved STI case management at a reasonable cost and should be widely used [54].

There are claims that overtreatment will result into drug resistance. It has been shown that the decreased susceptibility to ceftriaxone and spectinomycin is consistent with the widespread use of these agents in the community for other indications and there are doubts whether syndromic management or decrease of the total consumption of antibiotics by reverting to aetologic-based management of STIs will make any difference in the development of drug resistance in the absence of a more rational use of the same drugs for other indications [55].

Syndromic management has been shown to have the lowest programme cost compared to other approaches, while mass treatment is cost effective in terms of cost for cure. However the treatment seeking behaviour, STI prevalence and service coverage will determine the cost effectiveness of either syndromic approach, etiologic approach or mass treatment and will have impact on the programme [56, 57].

In resource poor settings, and until more rapid POC tests become available, syndromic approach remains to be an important option for managing symptomatic STIs. The trade-offs between trying to treat cervical STI through syndromic management, despite its poor performance, and deciding not to treat them need to be explicit. Behets and colleagues reviewed the implications of false- and true-positives and false- and true-negatives [19]. Low sensitivity results in high rates of missed treatment that can lead to persistence of infection that may result in complications and continued transmission, while a low specificity results in overtreatment leading to increased costs, adverse effects of drugs, and the negative consequence of being labelled as having an STI and a low PPV results in more false positives being treated unnecessarily as well as the psychosocial effect if being mislabelled as being infected.

In addition to diagnostic accuracy of the syndromic approach, the effectiveness could be enhanced by improvement of the quality of services and improving treatment seeking behaviours.

Based on this review and from previous review we conclude the vaginal discharge flowchart should focus on management of vaginal infection. We believe that the syndromic approach should be used as an intermediate approach for cervical infections for sex workers, since the prevalence of NG/CT infections amongst this population is higher and it’s better to lower the risk of transmission via this group, until a POC test is available in resource poor settings.

There are a number of limitations in the review presented. The studies included had varied clinical setting. Although all studies included women with symptoms of vaginal discharge, the prevalence of STIs and risk factors across the studies are varied. In addition, the majority of these studies have not been systematically recruited the study participants and thus may limit the generalizability of this review. Though the aggregated data did not measure odds ratio it did minimize variance of effect by weighted analysis. It should be noted that larger studies could influence the un-weighted estimates. Performance bias could be found in studies using a non-gold standard laboratory diagnostic tool as the test would have a lower accuracy rate [18]. Given the cross-sectional nature of studies, only associations can be inferred and no causal relationships can be determined. These studies have been conducted in a research environment where health care providers are trained and thus may overestimate the diagnostic accuracy of the flowcharts in actual health care setting.

## Appendix 1

PuBMed N = 303 'Vaginal discharge'[Mesh] OR “vaginal discharge” [TIAB] OR “vaginal discharges” [TIAB] OR “Leukorrhea” [all fields] OR “cervical discharge” [all fields] OR 'Cervix Uteri/secretion'[Mesh] OR 'cervical discharges' OR (vaginal AND discharge) OR (vagina and discharge) OR (cervix AND discharge) OR (cervix AND discharges) OR ‘vaginal secretion’ AND 'Software Design' OR flowcharts OR Flowchart OR algorithm OR algorithms OR 'flow charts' OR 'flow chart' OR 'clinical pathway' OR 'clinical pathways' OR 'risk assessment' OR syndromically OR syndromic OR signs OR symptoms OR symptom OR sign decision tree OR syndromic approach OR syndromic diagnosis OR syndromic management OR syndromic approaches

EMBASE N = 2436 'vagina discharge'/exp OR 'fluor vaginalis' OR ' genital fluor' OR 'vagina fluid' OR 'vagina fluor' OR 'vaginal discharge' OR 'vaginal fluid' OR 'vaginal fluor' OR 'leukorrhea' OR 'leukorrhea'/exp OR 'fluor albus' OR 'cervical discharges' OR (vaginal AND discharge) OR (vagina and discharge) OR (cervix AND discharge) OR (cervix AND discharges) OR ‘vaginal secretion’ OR ('uterine cervix '/exp AND (secretion OR discharge OR discharges OR secretions)) AND 'algorithm'/exp OR flowcharts OR Flowchart OR algorithm OR algorithms OR 'flow charts' OR 'flow chart' OR 'clinical pathway' OR 'clinical pathways' OR 'risk assessment' OR syndromically OR syndromic OR signs:ti,ab OR symptoms OR symptom OR sign:ti,ab OR 'decision tree' OR 'decision trees' OR 'syndromic approach' OR 'syndromic diagnosis' OR 'syndromic management' OR 'syndromic approaches'

POPLINE N = 18: VAGINAL ABNORMALITIES OR VAGINOSIS OR CERVICAL MUCUS OR CERVICAL EFFECTS AND SYNDROMIC MANAGEMENT OR SIGNS AND SYMPTOMS

Global Health Library N = 88:

Vaginal discharge AND syndromic approach OR flowchart.

## Supporting Information

S1 Checklist PRISMA 2009 Checklist. (DOC)
